# High GNG4 predicts adverse prognosis for osteosarcoma: Bioinformatics prediction and experimental verification

**DOI:** 10.3389/fonc.2023.991483

**Published:** 2023-02-10

**Authors:** Xiaohong Jiang, Fuxing Tang, Junlei Zhang, Mingwei He, Tianyu Xie, Haijun Tang, Jianhong Liu, Kai Luo, Shenglin Lu, Yun Liu, Jili Lu, Maolin He, Qingjun Wei

**Affiliations:** ^1^ Department of Trauma Orthopedic and Hand Surgery, The First Affiliated Hospital of Guangxi Medical University, Nanning, Guangxi, China; ^2^ Department of Orthopedic, The Affiliated Minzu Hospital of Guangxi Medical University, Nanning, Guangxi, China; ^3^ Department of Spinal Bone Disease, The First Affiliated Hospital of Guangxi Medical University, Nanning, Guangxi, China; ^4^ Department of Spinal Bone Disease, Yulin Orthopedics Hospital of Chinese and Western Medicine, Yulin, Guangxi, China; ^5^ Department of Sports Medicine, Southern University of Science and Technology Hospital, Shenzhen, Guangdong, China; ^6^ Department of Orthopaedics, the People’s Hospital of Baise, Baise, Guangxi, China

**Keywords:** GNG4, osteosarcoma, prognosis, biomarker, bioinformatics

## Abstract

**Background:**

Guanine nucleotide binding (G) protein subunit γ 4 (GNG4) is closely related to the malignant progression and poor prognosis of various tumours. However, its role and mechanism in osteosarcoma remain unclear. This study aimed to elucidate the biological role and prognostic value of GNG4 in osteosarcoma.

**Methods:**

Osteosarcoma samples in the GSE12865, GSE14359, GSE162454 and TARGET datasets were selected as the test cohorts. The expression level of GNG4 between normal and osteosarcoma was identified in GSE12865 and GSE14359. Based on the osteosarcoma single-cell RNA sequencing (scRNA-seq) dataset GSE162454, differential expression of GNG4 among cell subsets was identified at the single-cell level. As the external validation cohort, 58 osteosarcoma specimens from the First Affiliated Hospital of Guangxi Medical University were collected. Patients with osteosarcoma were divided into high- and low-GNG4 groups. The biological function of GNG4 was annotated using Gene Ontology, gene set enrichment analysis, gene expression correlation analysis and immune infiltration analysis. Kaplan–Meier survival analysis was conducted and receiver operating characteristic (ROC) curves were calculated to determine the reliability of GNG4 in predicting prognostic significance and diagnostic value. Functional *in vitro* experiments were performed to explore the function of GNG4 in osteosarcoma cells.

**Results:**

GNG4 was generally highly expressed in osteosarcoma. As an independent risk factor, high GNG4 was negatively correlated with both overall survival and event-free survival. Furthermore, GNG4 was a good diagnostic marker for osteosarcoma, with an area under the receiver operating characteristic curve (AUC) of more than 0.9. Functional analysis suggested that GNG4 may promote the occurrence of osteosarcoma by regulating ossification, B-cell activation, the cell cycle and the proportion of memory B cells. In *in vitro* experiments, silencing of GNG4 inhibited the viability, proliferation and invasion of osteosarcoma cells.

**Conclusion:**

Through bioinformatics analysis and experimental verification, high expression of GNG4 in osteosarcoma was identified as an oncogene and reliable biomarker for poor prognosis. This study helps to elucidate the significant potential of GNG4 in carcinogenesis and molecular targeted therapy for osteosarcoma.

## Introduction

Osteosarcoma (OS) is the most common primary malignant bone tumour among children and adolescents. It is characterized by the production of osteoid and immature bone from mesenchymal or osteoblast precursor cells (1, 2). OS is more common in the long epiphyses of the extremities, such as the distal femur, proximal tibia, and proximal humerus, and less common in the axial bones and elsewhere. OS is characterized by a high tendency for local invasion and early metastasis, with an overall 5-year survival rate of 50%–70% for patients with localized OS. However, if metastasis occurs at the time of diagnosis or as the disease progresses, the 5-year overall survival rate becomes less than 20% (3). Therefore, understanding the molecular mechanism of OS occurrence and development and finding new molecular therapeutic targets are urgent.

Guanine nucleotide-binding (G) proteins are regulators of transmembrane signalling pathways. G protein trimers are composed of α, β, and γ subunits and are responsible for conveying signals from G protein-coupled receptors (GPCRs) to a cell’s interior. The α subunit is typically the effector of GPCR activation, while the βγ heterodimer acts as the modulator of the signal (4, 5). G protein transmits information through a variety of signalling pathways, including the mitogen-activated protein kinase (MAPK), phosphoinositide 3-kinase (PI3K), and RhoGEF pathways (6). G proteins regulate cell metabolism, secretion, growth, proliferation, differentiation, and death (7). Many studies have shown the importance of G protein family members in cancer pathology. For example, G protein subunits β1 (GNB1) and γ2 (GNG2) are oncogenes (8, 9). Epigenetically silenced GNG7 promotes oesophageal, renal clear cell, and lung adenocarcinoma (10–12). GNG11 enhances gastric cancer cell adhesion, migration, and invasion (13). High GNG11 expression predicts poor ovarian cancer prognosis (14). GNG12 plays an important role in glioma, pancreatic cancer and OS. GNG12 overexpression inhibits tumour cell proliferation and migration (15–17). Thus, G protein family members may be biomarkers for tumour diagnosis and treatment.

GNG4, a key member of the γ subunits of G proteins, is located on chromosomes 1q43-q44 and plays an important role in the transmembrane system (18). In recent years, accumulated studies have investigated the role of GNG4 in tumours. Recent studies suggest that GNG4 expression is elevated in a variety of tumours, including colorectal, colon, gastric, lung adenocarcinoma, and gallbladder cancers, and is associated with poor prognosis in patients with these cancer types (19–24). GNG4 plays an important role in promoting tumour cell adhesion, migration, proliferation, and invasion by binding GPCRs (20, 22). Although these studies confirm the importance of high GNG4 expression in the development and progression of some tumours, the clinical significance and biological function of GNG4 in OS remain unclear. We are interested in whether GNG4 is also highly expressed in OS and whether it is associated with the development and prognosis of OS.

Therefore, this study intended to elucidate the biological role and molecular mechanism of GNG4 in OS. On the basis of the GEO and TARGET databases, we combined the gene expression matrix and clinical characteristics to evaluate the prognostic value of GNG4 and provide a reliable clinical reference for screening the adverse prognostic characteristics of patients with OS. Finally, we verified the expression of GNG4 in OS and its ability to predict survival and prognosis *via* reverse transcription quantitative real-time polymerase chain reaction (RT−qPCR) and immunohistochemistry. Moreover, the effect of GNG4 silencing on the viability, proliferation and invasion of OS cells was verified *in vitro*.

## Materials and methods

### Data collection

Two GEO cohorts, the GSE12865 and GSE14359 datasets, were screened from the GEO database (https://www.ncbi.nlm.nih.gov/). The gene expression data of GSE12865 (normal=2, tumour=12) is based on the platform of GPL6244, and that of GSE14359 (normal=2, tumour=18) is based on the platform of GPL96. The RNA sequencing data and clinical information of 84 osteosarcoma patients were acquired from the TARGET database (https://ocg.cancer.gov/programs/target). Gene expression data of musculoskeletal samples from 396 healthy humans were collected from the GTEx database (https://gtexportal.org/). The batch effects of the integrated GEO dataset (GSE12865 and GSE14359), as well as the GTEx +TARGET dataset (GTEx and TARGET databases), were eliminated by the combat algorithm from the “sva” R package (25). Pancancer RNA-seq data from the UCSC database (http://xena.ucsc.edu/) were used to verify the differential analysis of GNG4 expression in 33 human tumours. A family of G protein genes from the HUGO Gene Nomenclature Committee (HGNC) (https://www.genenames.org/), including 34 genes (**Supplementary Table 1**), was downloaded.

### Filtering of DEGs from the GEO dataset

The R software LIMMA package was used to screen for differentially expressed genes (DEGs) between normal and tumour samples of GSE12865 and GSE14359. The filtering criteria were set as follows: |log2-fold-change (FC)| >1, adjusted P value < 0.05. The upregulated and downregulated DEGs from these two GEO datasets were intersected with G protein family genes through an online Venn diagram (http://bioinformatics.psb.ugent.be/webtools/Venn/). According to the integrated GEO dataset and GTEx +TARGET dataset, we further identified the expression pattern and diagnostic value of GNG4 mRNA in OS.

### Analysis of the ScRNA-Seq transcript dataset

The scRNA-seq transcript dataset GSE162454, including 6 OS samples before chemotherapy, was obtained from the GEO database (https://www.ncbi.nlm.nih.gov/geo). The R package “Seurat” was used to perform the computational analysis. The Seurat “Merge” function was used to integrate the 6 OS samples. Quality control (200 <number of feature RNA <6000, percentage of mitochondrial genes <10%) was performed to filter low-quality cells. The R package “Harmony” was further used to eliminate the batch effect. Then, the Seurat “FindClusters” function was used to acquire the cell clusters, with the resolution set to 0.1. The Seurat “FindAllMarkers” function was used to find marker genes of the clusters, and the cell types were annotated based on the marker genes of each cluster. The expression and distribution of GNG4 were visualized using the Seurat “VlnPlot” and “FeaturePlot” functions.

### Survival correlation analysis

In the TARGET dataset, 84 patients with OS were divided into high-expression and low-expression clusters in accordance with the median value of GNG4 expression. The differences in overall survival and event-free survival (EFS) between the GNG4 high- and low-expression clusters were tested using the Kaplan–Meier method. The prediction efficiency of GNG4 was evaluated using the receiver operating characteristic (ROC) curve. Univariate and multivariate Cox regression analyses were performed to determine whether GNG4 was an independent prognostic factor. In addition, a prognostic nomogram was constructed using the R package “rms”. In the external validation cohort, the Kaplan−Meier method was used to analyse the survival outcomes of patients with high and low GNG4 expression, and univariate and multivariate Cox regression analyses were performed on the clinical characteristics and GNG4 expression of patients with OS.

### GNG4-related DEGs and functional enrichment analysis

Differentially expressed genes between high- and low-GNG4 expression clusters in the TARGET dataset were identified as GNG4-related DEGs. A difference analysis was performed using R software’s LIMMA package. The filtering criteria were as follows: |log2 FC|> 1 and adjusted P value < 0.05.

To better study the functional enrichment status of GNG4-related DEGs, gene ontology (GO) enrichment analysis was conducted using R software’s clusterProfiler package. Additionally, gene set enrichment analysis (GSEA) using R package clusterProfiler was conducted to elucidate the significant function and pathway between the high- and low-GNG4 groups. The thresholds were set to false discovery rate < 0.25, P value < 0.05, and |Nes|>1. In the GSEA, C2.cp.v7.2.symbols.gmt [curated] from MSigDB collections was used as the reference gene set.

### Protein–protein network construction and hub gene acquisition

We used Metascape online tools (https://metascape.org) to construct the PPI network of GNG4-related DEGs. The parameters were as follows: minimum value = 3 and maximum value = 500. To extract the key proteins in this PPI network, Molecular Complex Detection (MCODE; http://apps.cytoscape.org/apps/mcode), a plug-in for Cytoscape version 3.7.2 (https://cytoscape.org/), was used.

### Immune infiltration analysis

CIBERSORT (https://cibersort.stanford.edu/) and xCell algorithms were used to calculate immune cells in the sample of 84 OS cases and analyse the differences in immune cell infiltration between the GNG4 high- and low-expression clusters. We also determined whether any correlations existed between the two clusters of GNG4 expression in the infiltrated immune cells.

### Immunohistochemical assay

To further verify the efficacy of GNG4 expression in predicting survival, 58 tissue specimens (2 per case) were retrospectively collected from patients with OS at the First Affiliated Hospital of Guangxi Medical University with the approval of the Ethics Committee of the First Affiliated Hospital of Guangxi Medical University (2021 KY-E-041). Immunohistochemical staining of paraffin sections was performed in accordance with the standard protocol (anti-GNG4 antibody, Ab238868, 1:100). At least 100 tumour cells were detected in the 5 tissue regions with the strongest immune response to GNG4 antibodies *via* light microscopy with 100× and 400× microscopes. Patients were divided into high- and low-expression clusters in accordance with the GNG4 expression level. GNG4 positivity was assessed independently by two pathologists. Immunohistochemical results were assessed using a scoring system as described earlier (26). The product of the GNG4-positive rate and staining intensity was used to classify low GNG4 (0–4 points) and high GNG4 expression (>4).

### Cell culture

Human OS cells (143B, HOS, Saos, MG-63, U-2, and human osteoblast hFOB 1.19) were purchased from Fuheng Cell Center (Shanghai Fuheng Cell Center, China). HOS, Saos, MG-63 and hFOB 1.19 cells were cultured in Dulbecco’s modified Eagle’s medium (Gibco, USA). U-2 cells were cultured in McCoy’s 5A medium (Gibco, USA). 143B cells were cultured in 1640 medium (Gibco, USA). Human OS cells were cultured in a humidified 5% CO_2_ incubator at 37 °C. HFOB 1.19 cells were cultured in a humidified incubator at 33.5 °C and 5% CO_2_. The medium was supplemented with 1% penicillin/streptomycin (Solarbio, Beijing, China) and 10% foetal bovine serum (FBS; Gibco).

### Total RNA extraction and RT−qPCR

In accordance with the manufacturer’s instructions, total RNA was extracted using the Hipure Total RNA Mini Kit (Magen, China). RNA was reverse-transcribed into complementary DNA (cDNA) using a cDNA synthesis kit (Takara, Japan). RT−qPCR was performed using SYBR Green (FastStart Universal SYBR Green Master Mix (ROX, Germany). GAPDH (Abcam, USA) was used as a control. The normal GNG4 expression levels in five cell lines were expressed as relative expression and calculated using the 2-ΔΔCt method. The primer sequences of GNG4 mRNA were as follows: ‘5-GCATCTCCCAAGCCAGGAAAGC-3’ (F) and ‘5-GCAGGCactGGaATGATGAGAGG-3’ (R). Those of GAPDH were as follows: ‘5-CCCATCACCATCTTCCAGGAG-3’ (F) and ‘5-GTTGTCATGGATGACCTTGGC-3’ (R). All experiments were repeated three times.

### Transfection of cells

Silencing GNG4 (SiGNG4) RNA was designed and constructed by Sangon Biotechnology (Shanghai) Co., Ltd., and transfected into the U2 OS cell line using the manufacturer’s protocol. The transfection efficiency was measured according to the relative expression of GNG4. The SiGNG4 RNA: SiRNA99, SiRNA136, SiRNA218 and negative control (NC), SiRNA99: Sense-5’ -CCACUAGCAUCUCCCAAGCCATT-3 ‘and Antisense-5’ -UGGCUUGGGAGAUGCUAGUGGTT-3’, SiRNA136: Sense-5’ -GCUAAAGAUGGAAGCCUGUAUTT-3 ‘and Antisense-5’-AUACAGGCUUCCAUCUUUAGCTT-3’, SiRNA218: Sense-5’ -CGGGAAGAUCCUCUCAUCAUUTT-3 ‘and Antisense-5’-AAUGAUGAGAGGAUCUUCCCGTT-3’, Si-NC: Sense-5’-UUCUCCGAACGUGUCACGUTT-3’ and Antisense-5’-ACGUGACACGUUCGGAGAATT-3’. Transfected cells were obtained after transfection of U2 OS cells for 48 h.

### Cell viability assay

Cell viability was determined using a CCK-8 assay (Beyotime Institute of Biotech) following the manufacturer’s instructions. Herein, we seeded transfected U2 cells (2000 cells/well) into 96-well plates. Thereafter, 10% CCK-8 solution was added to each well and incubated in a dark environment at 37°C for 2 hours. Subsequently, cell proliferation was measured at 0, 24, 48, and 72 hours. The optical density (OD) of the cells at 450 nm was determined by a versatile fluorescent luminescence analyser (Varioskan LUX, Thermo Fisher).

### EDU cell proliferation assay

A BeyoClick™ EdU-488 cell proliferation assay kit (C0071S, Beyotime, Shanghai) was used to detect cell proliferation. Briefly, transfected U2 cells (2000 cells/well) were seeded in 96-well plates and then allowed to adhere. Cells were labelled with 100 μl/well of EDU solution for 2 hours and then fixed with 4% paraformaldehyde for 15 min. Subsequently, the cells were soaked alternately with closed solution and permeable solution 2 times (5 min each), and the cells were incubated with click staining solution for 30 min away from light. Finally, the click staining solution was removed, Hoechst solution was added after washing and the samples were incubated for 10 min away from light. The Hoechst solution was removed, and the cells were washed three times. Images were immediately taken using an inverted fluorescence phase contrast microscopy imaging analysis system (CellSens Dimension, OLYMPUS).

### Transwell invasion assay

The Matrigel matrix (356234, Corning, USA) was dissolved and diluted overnight at 4°C with serum-free media at a ratio of 1:3. Then, 50 μL of thinner was added to the base of the upper compartment. Transfected U2 cells (8×104/well) were inoculated into the upper chamber and treated with serum-free culture medium. Then, 500 µl of 10% foetal bovine serum medium was added to the lower chamber. The cells were incubated at 37°C for 48 h followed by fixation with 4% paraformaldehyde for 15 min. The cells on the upper membrane were wiped with cotton swabs and stained with 0.1% crystal indigo at room temperature for 10 min. Five microscope fields were randomly selected under a cellSens Dimension (OLYMPUS) for counting. The invasion ability of tumour cells was assessed by the number of cells entering the inferior lumen.

### Statistical analysis

Statistical analysis was performed using R (version 3.6.1). The Kruskal–Wallis test, Wilcoxon rank sum test, and chi-squared test were used to analyse the relationship between GNG4 expression and clinicopathological features. The survival curve was plotted using the Kaplan–Meier method, and the differences between groups were tested *via* a logarithmic sequence. ROC curves were generated using R software to evaluate the diagnostic performance of GNG4 expression. Univariate and multivariate Cox regression analyses were used to screen for independent prognostic factors. Significance was set at P value < 0.05.

## Results

### DEGs identified from the GEO dataset

**Figure 1:**
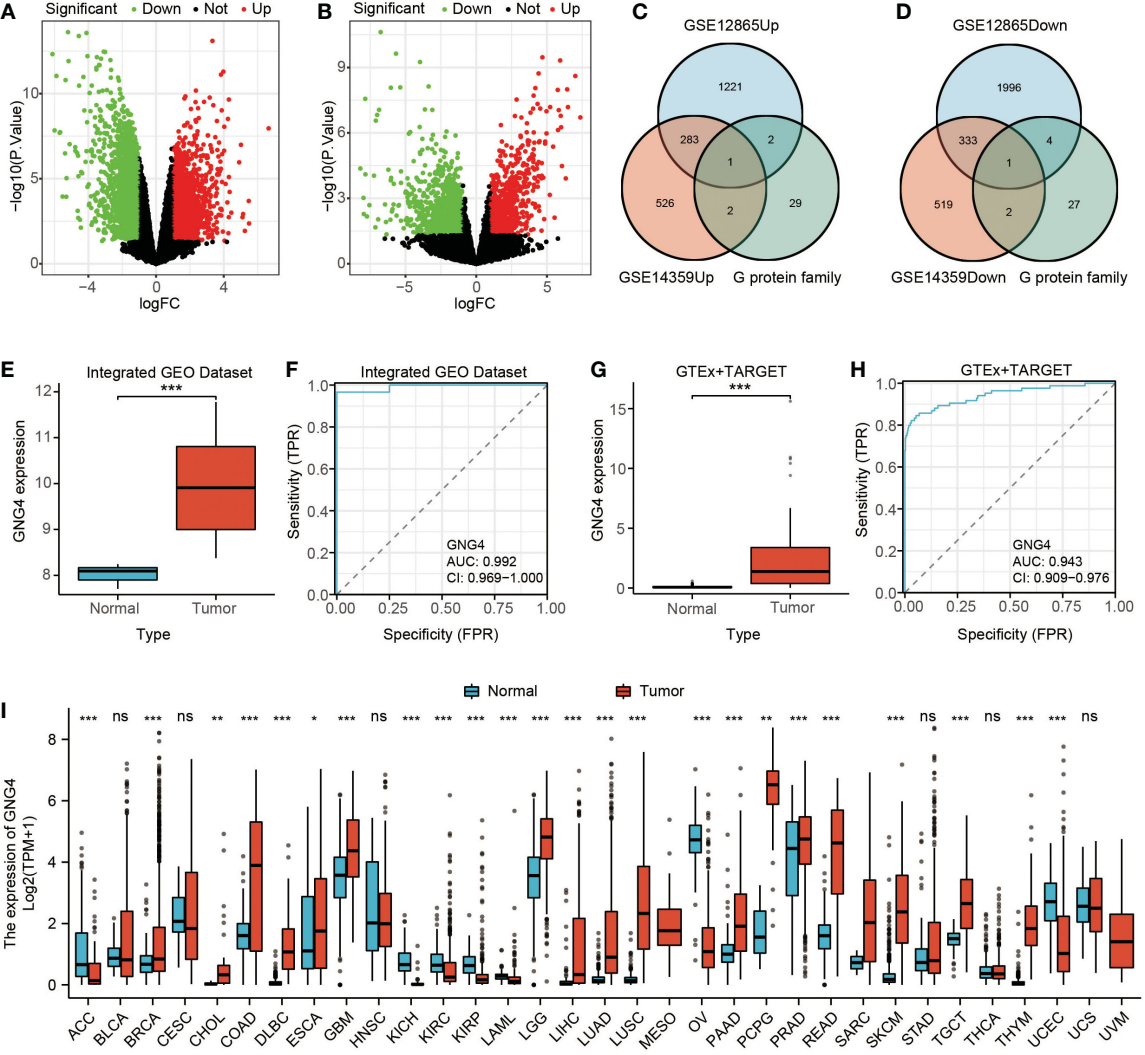
Filtered DEGs and GNG4 expression in OS. **(A, B)** The DEGs between normal and tumour samples of GSE12865 and GSE14359. The significant differences between the two groups are shown in the form of volcanic maps. **(C, D)** The G protein family genes were intersected with upregulated and downregulated genes in the two GEO datasets to obtain a Venn diagram of the intersected genes (upregulated GNG4 and downregulated GNG12). **(E, G)** GNG4 expression in OS in the integrated GEO dataset and GTEx + TARGET dataset, respectively. **(F, H)** The diagnostic ROC curve of GNG4 in the integrated GEO dataset and GTEx + TARGET dataset, respectively. **(I)** Differential expression of GNG4 in pancancer tissues. ***p<0.001, **p<0.01, *p≤0.05, and ns, p>0.05.

Through differential genetic analysis, 1,507 upregulated genes and 2,334 downregulated genes were found in GSE12865, while 812 upregulated genes and 855 downregulated genes were found in GSE14359. The data were visualized in volcanic form (**Figures 1A, B**).

### Expression analysis of GNG4 in OS and pancancer

The upregulated gene GNG4 and downregulated gene GNG12 were obtained through the intersection of the upregulated and downregulated genes with G protein family genes in these two GEO datasets (GSE12865 and GSE14359) *via* an online Venn diagram (**Figures 1C, D**). In both the integrated GEO dataset and the GTEx + TARGET dataset, the expression of GNG4 in osteosarcoma was significantly higher than that in normal samples (**Figures 1E–G**). Moreover, the area under the diagnostic ROC curve (AUC) of GNG4 in the integrated GEO dataset and GTEx + TARGET dataset was 0.992 and 0.943, respectively (**Figures 1F-H**). Pancancer RNA-Seq data were further used to verify the differential analysis of GNG4 expression in 33 human tumours. GN–G4 was highly expressed in 17 human malignancies, including adenoid cystic carcinoma (ACC), invasive breast cancer (BRCA), bile duct cell carcinoma (CHOL), and colonic adenocarcinoma (COAD) (**Figure 1I**).

### The scRNA-seq analysis

**Figure 2:**
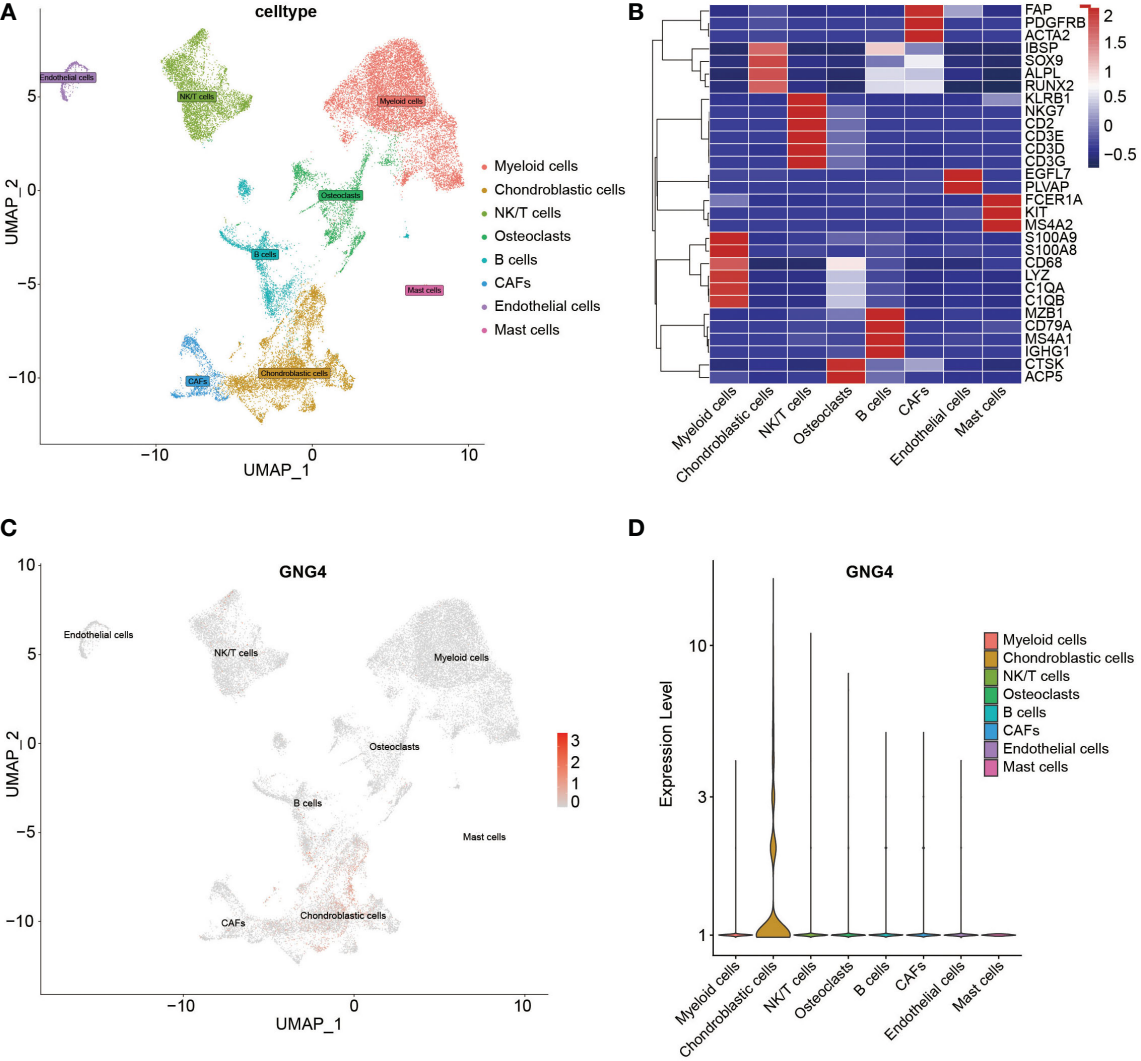
scRNA-seq analysis of the GSE162454 dataset. **(A)** UMAP plot of different cell types in the TME. **(B)** Heatmap of the marker genes of different cell types. **(C, D)** Feature plot and violin plot of GNG4.

With the existing cell types and corresponding marker genes as references, eight cell types were identified. As shown in **Figure 2A**, uniform manifold approximation and projection (UMAP) was used to classify and visualize the distribution and heterogeneity of the annotated eight cell types. The cell types and marker genes are shown in **Figure 2B**. Notably, GNG4 was highly expressed in chondroblastic cells but was expressed at low levels in other cell types (**Figures 2C, D**).

### Relationship between GNG4 expression and clinical parameters

In the evaluation of the relationship between GNG4 expression and various clinical indicators in the TARGET dataset, GNG4 expression was not associated with patient age, sex, metastasis, or tumour site, and high GNG4 expression was closely associated with relapse (**Table 1**).

**Table 1:** Relationship between GNG4 expression and clinical parameters in TARGET database.

Variable	Number of patients	GNG4 expression		
		Low	High	P value
		(N=42)	(N=42)	
Age				0.5027
>16 years	33 (39.3%)	18 (42.9%)	15(35.7%)	
≤16 years	51 (60.7%)	24(57.1%)	27 (64.3%)	
Gender				0.826
Female	37 (44.0%)	19 (45.2%)	18(42.9%)	
Male	47(56.0%)	23 (54.8%)	24 (57.1%)	
Relapse				**0.0002**
No	31 (44.9%)	22(68.8%)	9 (24.3%)	
Yes	38 (55.1%)	10 (31.2%)	28(75.7%)	
Metastasis				0.8011
No	63 (75.0%)	31 (73.8%)	32 (76.2%)	
Yes	21 (25.0%)	11 (26.2%)	10 (23.8%)	
Site				>0.9999
Arm/Hand	6 (7.1%)	3 (7.1%)	3 (7.1%)	
Leg/Foot	76 (90.5%)	38 (90.5%)	38 (90.5%)	
Pelvis	2 (2.4%)	1 (2.4%)	1 (2.4%)	

Values in bold indicate a P value less than 0.05, indicating a statistically significant difference.

### Prognostic value of GNG4 expression in OS

**Figure 3:**
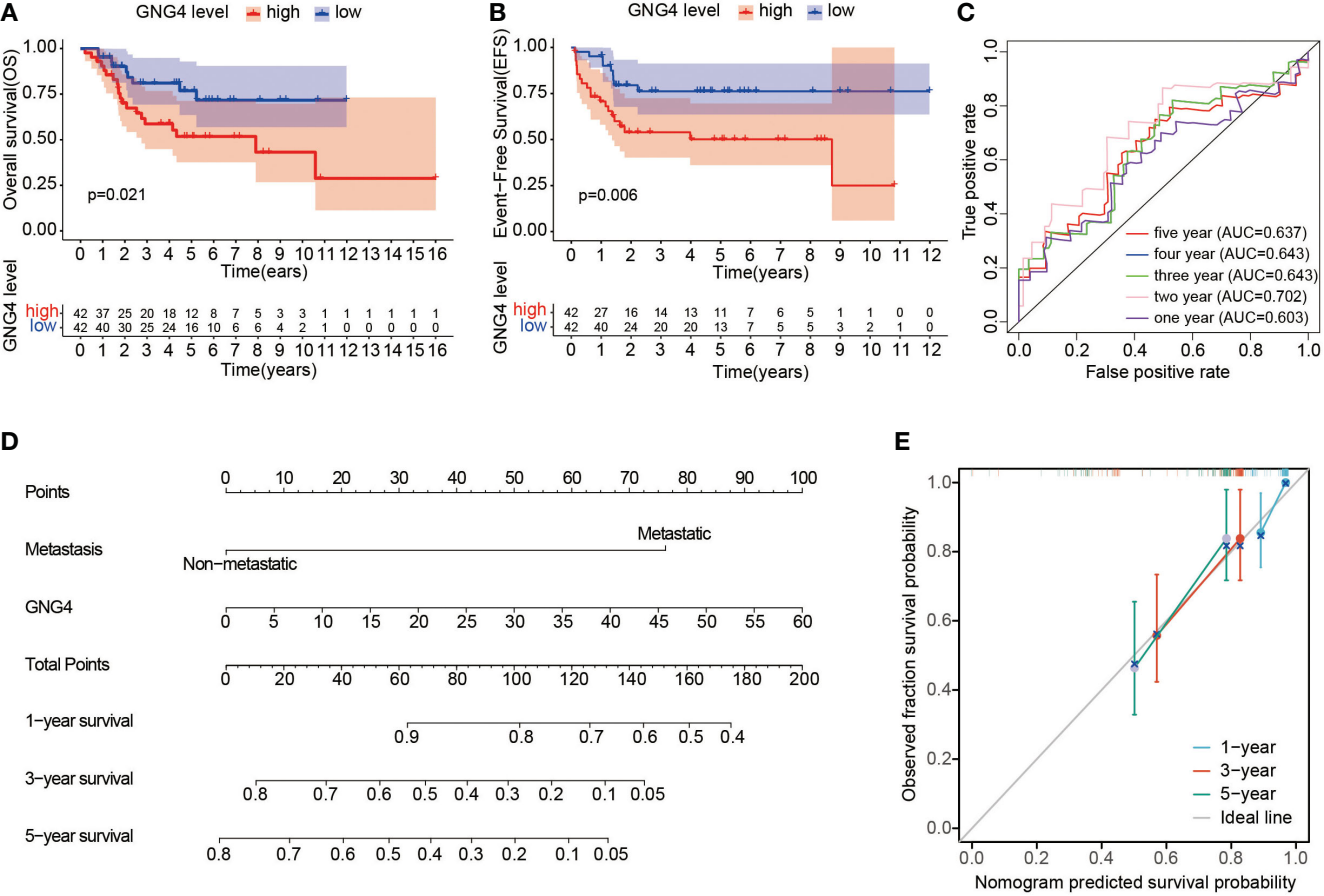
Prognostic value of GNG4 in OS. **(A, B)** In the TARGET dataset, patients with high GNG4 mRNA expression had worse overall survival time and EFS (n=84). **(C)** The AUC of GNG4 was determined in accordance with the time-dependent ROC curve. **(D)** Nomogram combining GNG4 and other prognostic factors. **(E)** Calibration curve of the nomogram.

In the TARGET dataset, 84 samples were divided into low- and high-expression clusters based on the median expression of GNG4 mRNA in OS (TPM 14.173). The Kaplan–Meier analysis showed that the overall survival and EFS were worse in the high-expression cluster than in the low-expression cluster (**Figures 3A, B**). The univariate and multivariate Cox regression analyses determined that metastasis and GNG4 were independent prognostic factors (**Table 2**). Analysis of the time-dependent ROC curves of GNG4 revealed that 1-/2-/3-/4-/5-year AUCs were 0.603/0.702/0.643/0.643 and 0.637, respectively (**Figure 3C**). Through time-dependent ROC curves, we found that GNG4 was a reliable predictor of patient prognosis. The prediction accuracy at 2 years was the highest, and its AUC value reached 0.702.

**Table 2:** Univariable and multivariable Cox regression analysis of clinical characteristics and GNG4 in TARGET database.

Variable	Univariate analysis			Multivariate analysis		
	HR	95%CI	P value	HR	95%CI	P value
GNG4	1.044	1.019-1.069	**<0.001**	1.037	1.012-1.063	**0.003**
High						
Low						
Age	0.828	0.384-1.786	0.63	1.227	0.513-2.935	0.646
>16 years						
≤16 years						
Gender	0.687	0.330-1.429	0.315	0.632	0.281-1.420	0.266
Female						
Male						
Metastasis	4.74	2.271-9.895	**<0.001**	4.309	2.016-9.211	**<0.001**
Yes						
No						
Site	2.372	0.490-11.495	0.283	3.075	0.510-18.521	0.22
Arm/Hand						
Leg/Foot						
Pelvis						

Values in bold indicate a P value less than 0.05, indicating a statistically significant difference.

### Construction of the clinical prediction model

To facilitate clinical prediction and evaluation, we constructed a clinical prediction model by fitting clinical parameters and GNG4 mRNA expression into the TARGET dataset. A nomogram was established to integrate the independent indicators, including GNG4 and metastasis. The sum of the corresponding score of each indicator is the total score. A higher total score in the nomogram indicates a worse prognosis. (**Figure 3D**). The calibration curve evaluated the performance of the GNG4 nomogram, with a concordance index of 0.800 (0.761-0.839) (**Figure 3E**). In conclusion, this nomogram may be a better model than a single prognostic factor for predicting survival in patients with OS.

### Identification of GNG4-related DEGs

**Figure 4:**
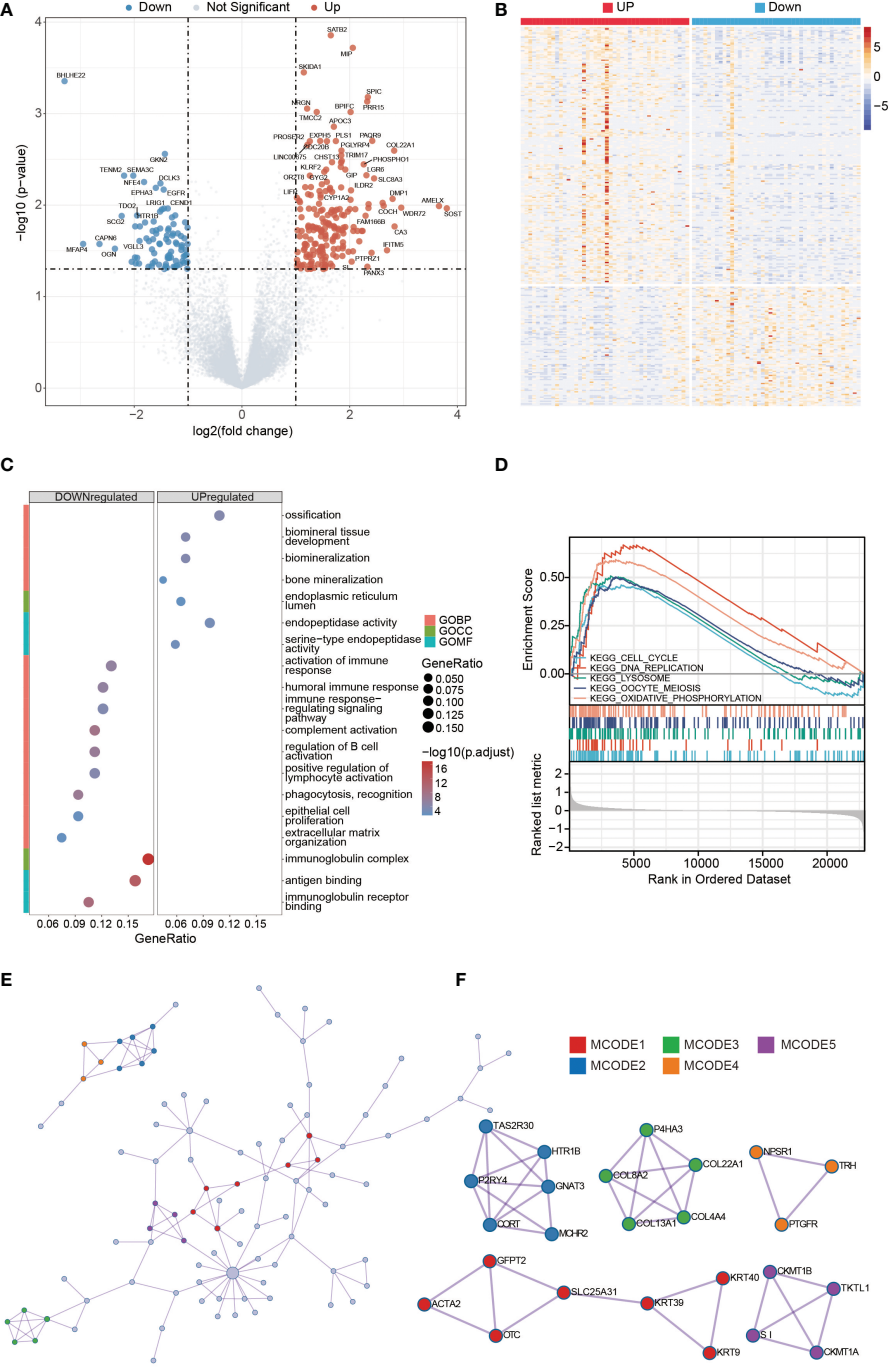
DEGs analysis of high- and low-GNG4 expression clusters. GO and GSEA functional enrichment analyses of GNG4, the PPI network, and hub gene cluster construction. **(A, B)** Grouped in accordance with the median GNG4 value in the TARGET dataset, a total of 224 significantly different genes were screened, including 127 upregulated and 97 downregulated genes. **(C)** The GO enrichment method was used to analyse the differentially expressed genes in the high- and low-GNG4 expression clusters. The bubble size represents the amount of gene concentration, and the colour represents the significance of the difference. **(D)** GSEA showed enriched genes involved in “ECM degradation,” “collagen,” “ECM glycoprotein,” “focal adhesion,” and “cell cycle” signalling pathways. **(E)** PPI networks from the TARGET dataset. **(F)** Five hub gene clusters were obtained from the TARGET dataset by using the MCODE clustering algorithm.

In the GNG4 low- and high-expression clusters of the TARGET dataset, 224 genes with significant differences were screened, including 127 upregulated and 97 downregulated genes. The data were visualized in the form of volcanoes and heatmaps (**Figures 4A, B**).

### Functional enrichment analysis

The results of the GO enrichment analysis in the TARGET dataset using R software indicate that the following terms were enriched. GNG4 upregulated DEGs were enriched in the BP terms: “ossification,” “biomineral tissue development,” “biomineralization” and “bone mineralization;” CC term: “endoplasmic reticulum lumen;” and MF terms: “endopeptidase activity” and “serine−type endopeptidase activity.” The downregulated DEGs were enriched in the BP terms: “activation of immune response,” “humoral immune response,” “immune response−regulating signalling pathway,” “complement activation,” “regulation of B-cell activation,” “positive regulation of lymphocyte activation,” “phagocytosis, recognition,” “epithelial cell proliferation” and “extracellular matrix organization;” CC term: “immunoglobulin complex;” and MF terms: “antigen binding” and “immunoglobulin receptor binding.” (**Figure 4C**). To further explore the key pathways related to GNG4, GSEA analysis was also performed. GSEA analysis results showed that “cell cycle,” “DNA replication,” “lysosome,” “oocyte meiosis,” and “oxidative phosphorylation” KEGG pathways were significantly enriched, indicating that these pathways may be involved in GNG4 carcinogenic mechanism (**Figure 4D**).

### PPI network and hub gene

Using the Metascape online tool, we built a PPI network from GNG4-related DEGs in the TARGET dataset (**Figure 4E**). Then, we used the MCODE clustering algorithm to screen out hub gene clusters (**Figure 4F**). In the TARGET dataset, the filtered hub genes are connected in the following networks: MCODE_1 (intermediate filament organization, intermediate filament cytoskeleton organization, and intermediate filament-based process; GFPT2, ACTA2, OTC, SLC25A31, KRT39, KRT9, and KRT40), MCODE_2 (G alpha (i) signalling events, GPCR downstream signalling, and signalling by GPCR; TAS2R30, HTR1B, GNAT3, MCHR2, CORT, and P2RY4), MCODE_3 (collagen biosynthesis and modifying enzymes, collagen formation, and NABA COLLAGENS; P4HA3, COL22A1, COL4A4, COL13A1, and COL8A2), MCODE_4 (digestion, and arginine and proline metabolism; CKMT1B, TKTL1, CKMT1A, and SI), MCODE_5 (G alpha (q) signalling events, neuroactive ligand−receptor interaction, and class A/1 (rhodopsin-like receptors); NPSR1, TRH, and PTGFR).

### Correlation analysis of immune infiltration

**Figure 5:**
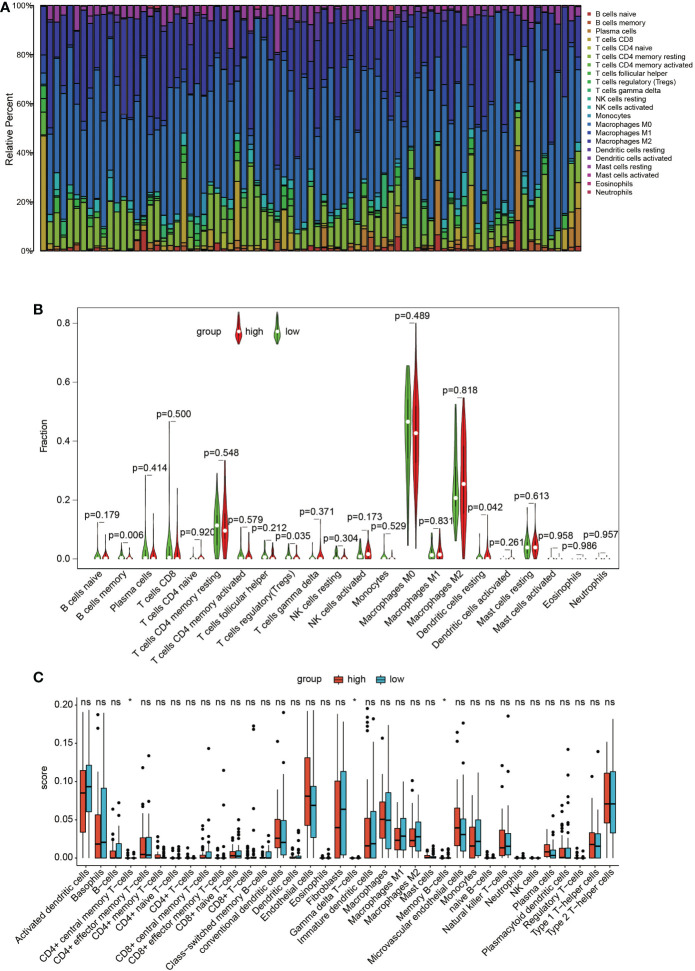
Immune infiltration. **(A)** Distribution of 22 types of immune cells in 84 OS samples. **(B)** Violin plot of 22 tumour-infiltrating immune cells in the low- and high-risk clusters by the CIBERSORT algorithm. **(C)** Boxplots of infiltrating immune cells in the low- and high-risk clusters by the xCell algorithm. p<0.05 was considered as statistical difference (*), ns as no statistical difference.

In the TARGET dataset, the distribution of the 22 immune cells was quite different in each sample (**Figure 5A**). The results of the CIBERSORT algorithm show that memory B cells and regulatory T cells (Tregs) in the cluster with high GNG4 expression were significantly lower than those in the cluster with low GNG4 expression, and resting dendritic cells were higher in the cluster with high GNG4 expression than in the cluster with low GNG4 expression (**Figure 5B**). The results of the xCell algorithm show that compared with the low GNG4 expression group, the high GNG4 expression group had significantly higher infiltration abundances of CD4^+^ central memory T cells and significantly lower infiltration abundances of gamma delta T cells and memory B cells (**Figure 5C**). The results of the CIBERSORT and xCell algorithms both show that high GNG4 expression was associated with low enrichment of memory B cells.

### Validating GNG4 expression and prognostic value

**Figure 6:**
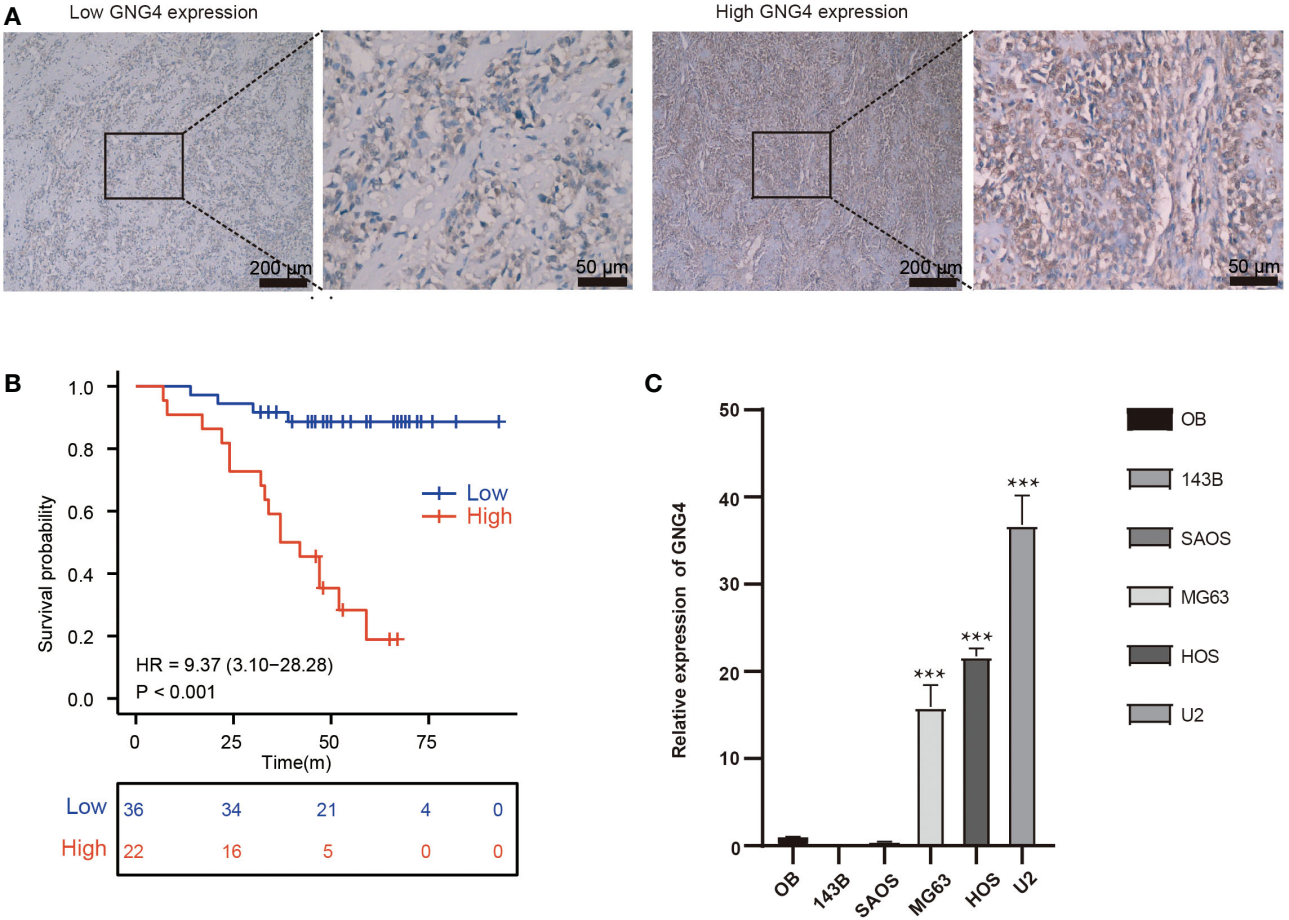
Verification of the expression and prognostic value of GNG4. **(A)** Immunocytochemical staining of GNG4 in OS tissues. **(B)** In the validation cohort, the effect of high GNG4 expression on overall survival prognosis was statistically significant. **(C)** GNG4 expression in hFOB cells and five OS cell lines. p<0.001 was considered as significant difference (***).

The expression of GNG4 protein in the tissues of 58 patients with OS was examined *via* immunohistochemistry to confirm the prognostic reliability of GNG4. Our results show that GNG4 was clearly localized in the cytoplasm of OS cells (**Figure 6A** **)**. Among the 58 OS tissues, GNG4 expression was high in 22 cases and low in 36 cases (**Table 3**). Next, the relationship between clinicopathological characteristics and GNG4 expression in OS patients was analysed. In general, the high expression of GNG4 was closely related to tumour relapse, metastasis and TNM stage in patients (**Table 3**). Univariate Cox regression analysis (**Table 4**) showed that GNG4 (HR=9.37, P < 0.001), relapse (HR=5.65, P < 0.001), metastasis (HR=10.69, P < 0.001) and TNM stage (HR=0.15, P < 0.001) were important factors in evaluating the prognosis of OS. The multivariate COX regression analysis (**Table 4**) suggested that GNG4 (HR=10.97, P < 0.002) and metastasis (HR=15.94, P < 0.02) were independent risk factors for overall survival. The results of the Kaplan–Meier analysis show that patients with high GNG4 expression had a poor survival prognosis (HR=9.37, P < 0.001) (**Figure 6B**). The expression of the GNG4 gene in OS cell lines (HOS, MG-63, and U-2) was higher than that in the hFOB cell line, and the differences among 143B, HOS, and MG-63 were statistically significant (P < 0.05) (**Figure 6C**). Taken together, these data suggest that GNG4 has important clinical significance in the prognosis and metastasis of patients with OS.

**Table 3:** Associations between clinicopathological characteristics and GNG4 in the external validation cohort.

Variable	Number of patients	GNG4 expression		
		Positive	Negative	P value
		(N=22)	(N=36)	
Age				0.309
>16 years	28 (48.3%)	13 (59.1%)	15 (41.7%)	
≤16 years	30 (51.7%)	9 (40.9%)	21 (58.3%)	
Gender				0.103
female	25 (43.1%)	6 (27.3%)	19 (52.8%)	
male	33 (56.9%)	16 (72.7%)	17 (47.2%)	
Relapse				**0.003**
No	43 (74.1%)	11 (50.0%)	32 (88.9%)	
Yes	15 (25.9%)	11 (50.0%)	4 (11.1%)	
Metastasis				**0.011**
No	37 (63.8%)	9 (40.9%)	28 (77.8%)	
Yes	21 (36.2%)	13 (59.1%)	8 (22.2%)	
TNM				**0.02**
I	22 (37.9%)	13 (59.1%)	9 (25.0%)	
II/III	36 (62.1%)	9 (40.9%)	27 (75.0%)	
Site				0.417
Else	9 (15.5%)	5 (22.7%)	4 (11.1%)	
Femur/Tibia	49 (84.5%)	17 (77.3%)	32 (88.9%)	
Size				0.417
>6 cm	29 (50.0%)	13 (59.1%)	16 (44.4%)	
≤6 cm	29 (50.0%)	9 (40.9%)	20 (55.6%)	

Values in bold indicate a P value less than 0.05, indicating a statistically significant difference.

**Table 4:** Univariable and multivariable Cox regression analysis of clinical characteristics and GNG4 in the external validation cohort.

Variable	Univariate analysis			Multivariate analysis		
	HR	95%CI	P value	HR	95%CI	P value
GNG4	9.37	3.1-28.28	**0.00007**	10.97	2.43-49.43	**0.002**
Positive						
Negative						
Age	1.42	0.59-3.43	0.43764			
>16 years						
≤16 years						
Gender	0.76	0.31-1.83	0.53589			
Female						
Male						
Relapse	5.65	2.3-13.91	**0.00016**	1.25	0.36-4.35	0.726
Yes						
No						
Metastasis	10.69	3.54-32.22	**0.00003**	15.94	1.55-163.61	**0.02**
Yes						
No						
TNM stage	0.15	0.05-0.41	**0.00023**	3.35	0.42-26.77	0.253
I						
II/III						
Location	0.49	0.18-1.36	0.17094			
Femur/Tibia						
Else						
Tumor size	0.49	0.2-1.23	0.12952			
>6 cm						
≤6 cm						

Values in bold indicate a P value less than 0.05, indicating a statistically significant difference.

### GNG4 function *in vitro*

**Figure 7:**
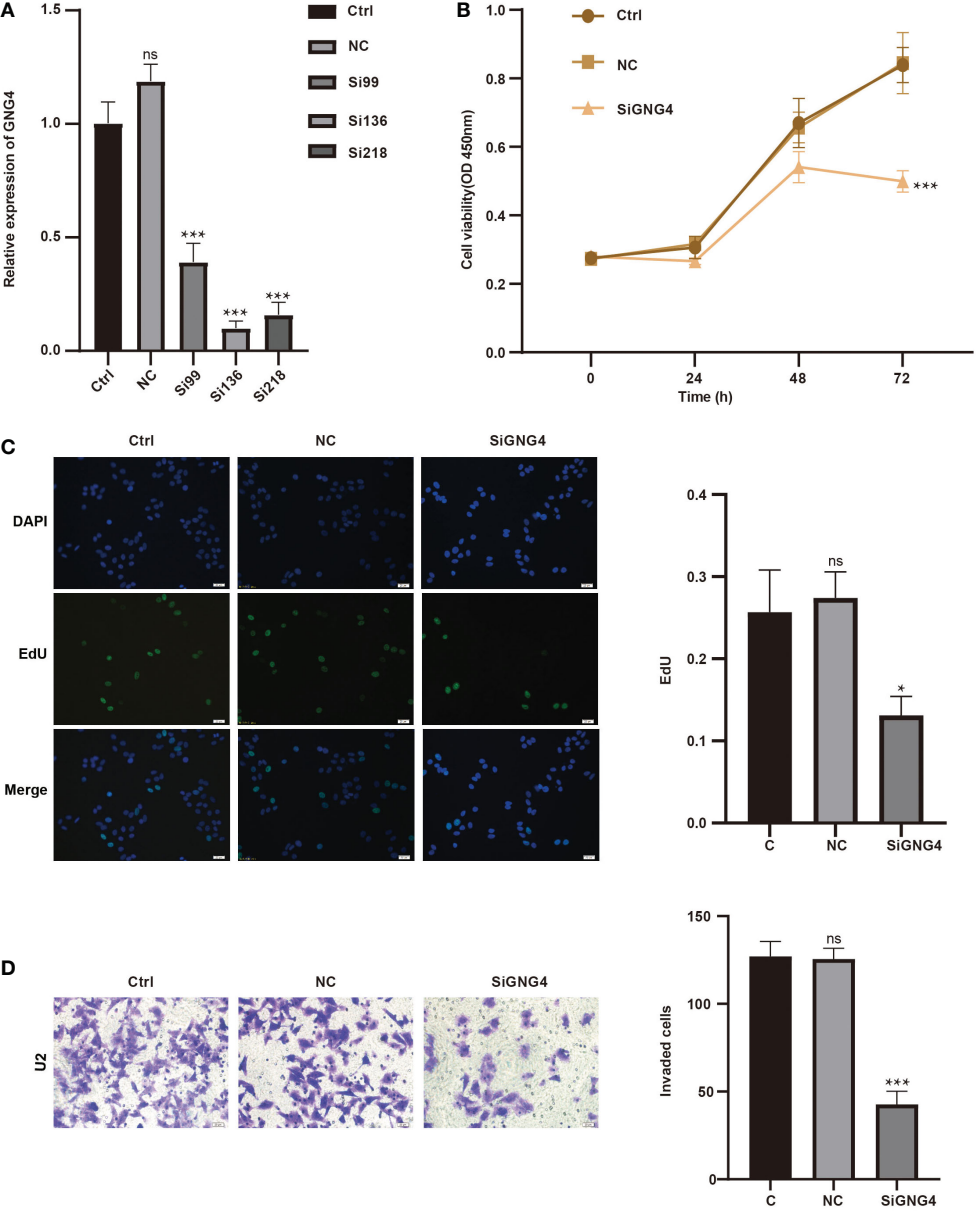
GNG4 function *in vitro*. **(A)** RT−PCR was used to verify the efficiency of GNG4 silencing. **(B)** OS cell viability assay after silencing GNG4. **(C)** EdU assay of OS cells after GNG4 silencing. **(D)** Invasion assay of OS cells after GNG4 silencing. p<0.001 was considered as significant difference (***) p≤0.05 was considered as statistical difference (*), ns as no statistical difference.

To analyse GNG4 function *in vitro*, we first constructed the SiGNG4 RNA: SiRNA99, SiRNA136, SiRNA218 and negative control (NC) and then transfected U2 cells. RT−PCR showed that the expression levels of GNG4 were significantly reduced after transfection of SiRNA99, SiRNA136 and SiRNA218 (**Figure 7A**). SiRNA136 was selected in the subsequent experiment to silence GNG4 expression because it had the best silencing efficiency.

Then, to verify the functional changes in U2 cells after GNG4 silencing, three groups were established: the control group, NC group, and SiGNG4 group. The results of CCK-8 analysis show that SiGNG4 decreased the activity of U2 cells at 24, 48 and 72 h after transfection (**Figure 7B**). In addition, compared to the control group and NC group, we observed a significant decrease in EdU-positive U2 cells after SiGNG4 treatment (**Figure 7C**). The Transwell invasion results show that, compared to the control group and NC group, the invasion ability in the SiGNG4 group was significantly reduced (**Figure 7D**). In conclusion, SiGNG4 can inhibit the viability, proliferation and invasion of OS cells.

## Discussion

Given its high heterogeneity, the overall survival rate of OS is not ideal (3). Therefore, effective and accurate evaluation of the prognosis of OS is highly significant. In the present study, DEGs between normal and osteosarcoma samples were screened through OS chip data from the GEO database, and then DEGs related to G protein were obtained *via* intersection with G protein family genes: G protein subunit γ 12 (GNG12) (low expression) and G protein subunit γ 4 (GNG4) (high expression), which are both γ subunits of the G protein. A previous study showed that GNG12 is a potential prognostic biomarker and a potential immunotherapy target in OS (17). However, the role of GNG4 in OS remains unknown.

Through bioinformatics and immunohistochemistry analysis, we determined that GNG4 mRNA and protein expression were associated with the overall survival time and EFS of OS. Moreover, the Cox regression analysis suggested that GNG4 could be an independent prognostic factor of OS. The functional enrichment analysis showed that the expression of GNG4 was related to multiple cancer signalling pathways and immune cell infiltration in OS. Therefore, our study illustrates the potential role of GNG4 in the pathogenesis of OS and establishes a foundation for further research.

Many previous studies have shown that GNG4 may be a diagnostic marker for various cancers, and GNG4 is highly expressed in different types of cancers, including rectal, colon, stomach, lung adenocarcinoma, and gallbladder cancers (19–24). In the TCGA data, our analysis also confirmed that GNG4 was significantly overexpressed in OS and most other tumours. Additionally, GNG4 was mainly expressed in chondroblastic cells according to the scRNA-seq analysis. This further implies that the GNG4 gene may be involved in the occurrence and development of OS. Our analysis also confirmed that GNG4 expression was a good diagnostic marker for OS, with an AUC greater than 0.9. The above data indicate that GNG4 not only serves as an oncogene in osteosarcoma but is also an excellent biomarker for distinguishing osteosarcoma from normal samples.

High GNG4 expression in OS is associated with poor prognosis. In accordance with the TARGET dataset, patients with high GNG4 expression had worse overall survival time and EFS. High GNG4 expression in OS is associated with tumour progression. The Cox regression analysis data indicate that metastasis and GNG4 were independent prognostic factors for the survival time. The above results were also verified in the external validation cohort. These results suggest that GNG4 expression is a prognostic biomarker of OS. Although many studies have suggested that GNG4 may be a biomarker for poor prognosis in a variety of tumours (19–24), this study is the first to investigate the correlation between GNG4 expression and prognosis in OS. Considering that metastasis and GNG4 are strong prognostic factors for OS, a nomogram was constructed that combined metastasis and GNG4 expression with clinical data. Nomograms can more accurately predict the overall survival of patients with OS for 1-/3-/5-year prognoses to help screen patients at high risk and provide an opportunity to identify more aggressive treatment options for patients at high risk. The calibration curve further verifies the validity of the nomogram.

We explored the potential biological function of GNG4 in OS through GO enrichment analyses of GNG4-related DEGs. GNG4-upregulated DEGs were determined to be enriched in ossification (27, 28), mineralization (29, 30), endopeptidase activity (31, 32), and endoplasmic reticulum lumen (33, 34). These findings indicate that GNG4 is involved in bone mineralization associated with the progression of OS. The regulation of endoplasmic reticulum function plays an important role in the treatment of OS (33, 34). The downregulated DEGs were enriched in the immune response (35), regulation of B-cell activation (36), and positive regulation of lymphocyte activation (37). These findings indicate that GNG4 is closely related to immunosuppression in OS. GNG4-related DEGs were enriched in cell cycle (38), DNA replication (39), lysosome (40) and oxidative phosphorylation (41, 42) in the GSEA enrichment analyses. The results of the GSEA enrichment analyses indicate that GNG4 was closely related to the cell cycle and proliferation pathways of osteosarcoma cells, which was confirmed by *in vitro* experiments. Through the PPI network, we identified five possible hub gene clusters associated with OS. These gene clusters affect the proliferation, adhesion, migration, and invasion of OS cells and participate in the progression of OS (43–45). In conclusion, our study provides insight into the role of GNG4 in the pathogenesis of OS and demonstrates that GNG4 is a potential biomarker and molecular therapeutic target for OS.

Immune cell infiltration in tumours is related to tumour progression and prognosis and contributes to the development of new therapeutic strategies (46). GNG4 has been reported to be associated with tumour immunity (20). Here, we investigated the relationship between GNG4 and the immune infiltration of OS through two immune infiltration algorithms. The immune infiltration analysis of the TARGET dataset showed that high GNG4 expression is associated with low enrichment of memory B cells. Memory B cells have been documented as a predictor of excellent patient survival in gastric cancer, head and neck squamous cell carcinoma, and colorectal cancer. For example, memory B cells have a significant effect on gastric cancer progression and prognosis, and higher levels of memory B cells reflect better overall survival (47). Other evidence suggested that a high density of memory B cells in HNSCC could predict an increased prognosis, and CD4+ T cells might affect B lymphocytes and their subsets through the CXCL13/CXCR5 axis (48). Furthermore, novel immunotherapy could potentially target memory B cells to shape the tumour microenvironment to repress tumourigenesis. Recent research has revealed that IgG1 memory B cells can reconstruct the tumour immune microenvironment and mobilize T cells and DCs to boost the immune machinery for tumour cell killing, thus providing insightful clues about the adoptive transfer of memory B cells in tumour immunotherapy (49). Based on previous studies, we speculated that GNG4 might inhibit memory B cells, thus promoting the occurrence and development of osteosarcoma. This is consistent with the functional enrichment analysis results that indicate high GNG4 expression suppressed the immune response.

This study further validated that OS patients with high GNG4 expression had a worse prognosis through analysis of their own clinical data cohort. *In vitro*, we found that GNG4 was highly expressed in osteosarcoma cells. After silencing GNG4, the viability, proliferation and invasion of OS cells were significantly inhibited. In conclusion, GNG4 can be used as a prognostic biomarker as well as a potential target for the treatment of OS.

Our study has some limitations. (1) The sample size of OS patients was considerably larger than that of the healthy controls. In future studies, we hope to increase the sample size of the control group. (2) Our results were validated in a cohort of 58 patients with OS; however, this study was a retrospective analysis. Therefore, prospective methods should be adopted in future studies to avoid analytical bias. (3) Although we demonstrated that GNG4 is a potential biomarker for OS, its underlying molecular mechanism should be further validated *in vivo* and *in vitro*.

In conclusion, this study confirmed that GNG4 is a potential biomarker for predicting the prognosis of OS. High expression of GNG4 in OS is associated with poor prognosis and can be used as an independent prognostic factor. Bioinformatics analysis indicated that GNG4 may be involved in the biological function of OS by regulating “ossification,” “mineralization,” the “immune response,” “endoplasmic reticulum lumen,” and “cell cycle”. Immune infiltration analysis suggested that GNG4 may influence the tumour microenvironment by regulating the proportion of memory B cells. Finally, we confirmed the feasibility of GNG4 as a prognostic biomarker and potential therapeutic target *in vitro* and in an external validation cohort. These findings provide a new perspective for the application of GNG4 as a potential biomarker and molecular therapeutic target for OS.

## Data availability statement

The original contributions presented in the study are included in the article/**Supplementary Material**. Further inquiries can be directed to the corresponding authors.

## Ethics statement

The studies involving human participants were reviewed and approved by the Ethics Committee of the First Affiliated Hospital of Guangxi Medical University(2021 KY-E-041. Written informed consent to participate in this study was provided by the participants’ legal guardian/next of kin.

## Author contributions

QW, MaH and JLu designed and supervised the research activity planning. XJ, and FT drafted the manuscript. JZ, MiH, TX, HT, JL, KL, SL and YL collected data and revised the manuscript. All authors contributed to the article and approved the submitted version.
